# Dietary fructose causes defective insulin signalling and ceramide accumulation in the liver that can be reversed by gut microbiota modulation

**DOI:** 10.1080/16546628.2017.1331657

**Published:** 2017-06-09

**Authors:** Raffaella Crescenzo, Arianna Mazzoli, Blanda Di Luccia, Francesca Bianco, Rosa Cancelliere, Luisa Cigliano, Giovanna Liverini, Loredana Baccigalupi, Susanna Iossa

**Affiliations:** ^a^ Department of Biology, Federico II University of Naples, Naples, Italy

**Keywords:** Fructose, insulin, obesity, inflammation, microbiota

## Abstract

**Objective**: The link between metabolic derangement of the gut–2013liver–visceral white adipose tissue (v-WAT) axis and gut microbiota was investigated.

**Methods**: Rats were fed a fructose-rich diet and treated with an antibiotic mix. Inflammation was measured in portal plasma, ileum, liver, and v-WAT, while insulin signalling was analysed by measuring levels of phosphorylated kinase Akt. The function and oxidative status of hepatic mitochondria and caecal microbiota composition were also evaluated.

**Results**: Ileal inflammation, increase in plasma transaminases, plasma peroxidised lipids, portal concentrations of tumour necrosis factor alpha, lipopolysaccharide, and non-esterified fatty acids, were induced by fructose and were reversed by antibiotic. The increased hepatic ceramide content, inflammation and decreased insulin signaling in liver and v-WAT induced by fructose was reversed by antibiotic. Antibiotic also blunted the increase in hepatic mitochondrial efficiency and oxidative damage of rats fed fructose-rich diet. Three genera, Coprococcus, Ruminococcus, and Clostridium, significantly increased, while the Clostridiaceae family significantly decreased in rats fed a fructose-rich diet, and antibiotic abolished these variations

**Conclusions**: When gut microbiota modulation by fructose is prevented by antibiotic, inflammatory flow from the gut to the liver and v-WAT are reversed.

## Introduction

Nutrient intake is a fundamental determinant of health. Many studies have correlated excess fructose intake with detrimental health outcomes, such as the metabolic syndrome [1,2]. Accordingly, using adult rats as an animal model, we have previously shown that obesity and insulin resistance are elicited by a fructose-rich diet [3–7]. In the past decade, a body of literature has reported that some components of the diet could exert a profound influence on the composition of gut microbiota, which has been shown to play a primary role in maintaining health. Changes in the composition of the gut microbiota induced by a high-fat diet are involved in the onset of obesity and the accompanying low-grade inflammation [8]. On the other hand, less is known about the putative link between the gut microbiota and fructose-rich diets. It has been proposed that continuous exposure to fructose may cause dysbiosis, with loss of microbial genetic and phylogenic diversity, promoting the evolution and maintenance of a ‘Western’ gut microbiome [9].

We have shown that antibiotics or faecal transplantation [10] can partly reverse fructose-induced altered whole-body glucose homeostasis and insulin resistance in skeletal muscle, as well as increased systemic plasma proinflammatory molecules, lipopolysaccharide (LPS), and tumour necrosis factor-α (TNF-α). LPS is a gut-derived endotoxin and therefore its increase in fructose-fed rats suggests a condition of inflammation in the gut induced by fructose. Moreover, LPS from the intestine reaches the general circulation through the portal system, and therefore the first tissue exposed to this proinflammatory molecule is the liver. The anatomic site of the liver places it in a strategic buffering position for absorbed fructose, and also for other substances coming from the intestine.

To highlight the link between the gut microbiota and fructose-induced metabolic derangement of the gut–liver–visceral white adipose tissue (v-WAT) axis, we used antibiotic administration as an experimental approach [11–14] to modulate populations of gut bacteria.

Adult rats were fed a fructose-rich diet in combination with antibiotic mix for 8 weeks. The development of hepatic insulin resistance; the degree of tissue inflammation in ileum, liver, and v-WAT; and portal inflammatory parameters were evaluated in comparison with values found in rats fed a fructose-rich diet only. The oxidative function and oxidative status of hepatic mitochondria were also assessed in control and treated rats. Metagenomic analysis of gut microbiota was carried out in the search for a correlation between changes in selected bacteria and altered hepatic metabolism.

The results clearly indicate that, when modulation of gut microbiota by dietary fructose is overcome by antibiotic administration, inflammatory flow from the gut to the liver and v-WAT, together with insulin resistance in the liver and v-WAT, as well as hepatic mitochondrial derangement, can be reversed.

## Methods

### Animals and treatments

Male Sprague–Dawley rats (Charles River, Calco, Lecco, Italy), about 100 days old, were caged singly in a temperature-controlled room (23 ± 1°C) with a 12 h light/dark cycle (06.30–18.30 h). Treatment, housing, and killing of animals met the guidelines set by the Italian Health Ministry. All experimental procedures involving animals were approved by the Comitato Etico-Scientifico per la Sperimentazione Animale of the University ‘Federico II’ of Naples.

Rats were divided into two groups and fed a fructose-rich or a control diet for 8 weeks. The composition of the two diets is shown in Table 1. Rats were pair-fed for the whole experimental period, being given the same amount of diet, both as weight and as caloric content, and each rat consumed the full portion of the diet. Half of the rats in each group also received broad-spectrum antibiotics [ampicillin (1.0 g/l) and neomycin (0.5 g/l)] in drinking water, while the other half of the rats received plain water. As ampicillin and neomycin are poorly absorbed, such treatment primarily affects only the intestinal microbiota, without direct systemic effects [15]. During the treatment, body weight, food, and water intake were monitored daily. Faeces were also collected daily and their energy content was assessed with a bomb calorimeter.

**Table 1.** Composition of experimental diets.

	Control diet	Fructose diet
Component (g/100 g)		
Standard chow^a^	50.5	50.5
Sunflower oil	1.5	1.5
Casein	9.2	9.2
Alphacel	9.8	9.8
Starch	20.4	–
Fructose	–	20.4
Water	6.4	6.4
AIN-76 mineral mix	1.6	1.6
AIN-76 vitamin mix	0.4	0.4
Choline	0.1	0.1
Methionine	0.1	0.1
Gross energy density (kJ/g)	17.2	17.2
Metabolizable energy density (kJ/g)^b^	11.1	11.1
Protein (% metabolizable energy)	29.0	29.0
Lipids (% metabolizable energy)	10.6	10.6
Carbohydrates (% metabolizable energy)	60.4	60.4
Of which:		
o Fructose	–	30.0
o Starch	52.8	22.8
o Sugars	7.6	7.6

^a ^Mucedola 4RF21 (Italy).

^b ^Estimated by computation using values (kJ/g) for energy content as follows: protein 16.736, lipid 37.656, and carbohydrate 16.736.

At the end of the experimental period, the rats were killed by decapitation; portal blood, systemic blood, caecum, and liver, as well as small samples of ileum and mesenteric v-WAT, were collected; and the carcasses were used for determination of body composition.

### Glucose tolerance test, plasma transaminases, triglycerides, and lipid peroxidation

A glucose tolerance test was carried out after 4 and 8 weeks of treatment. Rats were fasted for 6 h from 09.00 h. A basal, postabsorptive blood sample was obtained from a small tail clip and placed in ethylenediaminetetraacetic acid (EDTA)-coated tubes, and then glucose (2 g/kg body weight) was injected intraperitoneally. Blood samples were collected after 15 and 30 min and placed in EDTA-coated tubes. The blood samples were centrifuged at 1400 x *g*_av_ for 8 min at 4°C. Plasma glucose concentration was measured by a colorimetric enzymic method (Pokler Italia, Genova, Italy), while plasma insulin concentration was measured using an enzyme-linked immunosorbent assay (ELISA) kit (Mercodia, Uppsala, Sweden) in a single assay to remove interassay variations. Basal postabsorptive values of plasma glucose and insulin were used to calculate the homeostatic model assessment (HOMA) index, as: (Glucose (mg/dl) x Insulin (mU/l))/405 [16]. The magnitude of the rise in plasma glucose and insulin concentrations immediately (0–30 min) following the glucose load is proportional to the magnitude of hepatic insulin resistance [17]. Therefore, we calculated both the glucose area under the curve (AUC) and insulin AUC during the first 30 min after the glucose load, and the product (AUC glucose × AUC insulin) was used as an indirect index of hepatic insulin resistance [17].

Plasma concentrations of alanine aminotransferase (ALT), aspartate aminotransferase (AST), and triglycerides were measured by a colorimetric enzymic method using commercial kits (SGM Italia, Roma, Italy). Lipid peroxidation was determined according to Fernandes et al. [18], by measuring thiobarbituric acid reactive substances (TBARS) using the thiobarbituric acid assay. Aliquots of plasma were added to 0.3 ml of ice-cold 40% trichloroacetic acid. Then, 1 ml of 0.67% of aqueous thiobarbituric acid containing 0.01% of 2,6-di-tert-butyl-*p*-cresol was added. The mixtures were heated at 90°C for 15 min, then cooled in ice for 10 min, and centrifuged at 850 x *g* for 10 min. The supernatant fractions were collected and lipid peroxidation was estimated spectrophotometrically at 530 nm. The amount of TBARS formed was calculated using a molar extinction coefficient of 1.56 × 10^5^/mol/cm and expressed as nmol TBARS/ml.

### Portal plasma non-esterified fatty acids (NEFAs), LPS, and TNF-α levels

Portal plasma NEFA levels were measured by a colorimetric enzymic method (Roche Diagnostics, Mannheim, Germany). Portal plasma LPS was determined using a kit based on a *Limulus amaebocyte lysate*(LAL) extract (Lonza, Basel, Switzerland). In brief, samples were mixed with the LAL reagent and chromogenic substrate reagent for 16 min, and absorbance readings were taken on a plate reader at 405 nm.

TNF-α concentrations in portal plasma samples and in protein extracts from the ileum were determined using a rat-specific ELISA (R&D Systems, Minneapolis, MN, USA) according to the manufacturer’s instructions. In brief, the wells of a microtitre plate were coated with 100 µl of mouse anti-rat TNF-α (4 µg/ml) in phosphate-buffered saline (PBS) (137 mM NaCl, 2.7 mM KCl, 8.1 mM Na_2_HPO_4_, 1.5 mM KH_2_PO_4_, pH 7.4) and incubated overnight at room temperature. The antibody excess was then removed by washing with Wash Buffer [containing 0.05% (v/v) Tween 20 in PBS, pH 7.4], and the remaining sites on the plate were blocked with reagent diluent [PBS containing 1% bovine serum albumin (BSA)] (1 h, room temperature). After extensive washing, 100 μl of samples (1:10 dilution in reagent diluent) were added to the wells and incubated for 2 h at room temperature. After further washing, the wells were incubated with biotinylated goat anti-rat TNF-α (225 ng/ml in reagent diluent) followed by treatment with streptavidin–horseradish peroxidase (HRP) (1:200 dilution; 1 h, room temperature). Peroxidase-catalysed colour development from *o*-phenylenediamine was measured at 492 nm.

### Body composition, liver composition, and energy balance

Guts were cleaned of undigested food and the carcasses were then autoclaved. After dilution in distilled water and subsequent homogenization of the carcasses with a Polytron homogenizer (Kinematica, Luzern, Switzerland), duplicate samples of the homogenized carcass were analysed for energy content by a bomb calorimeter. The contribution of the liver to total body energy content was determined by measuring the energy content of liver samples with the bomb calorimeter. Total body water content was determined by drying carcass samples in an oven at 70°C for 48 h. Total body lipids were measured by the Folch extraction method [19]. The energy as lipid was calculated from body lipids using the coefficient of 39.2 kJ/g, and was then subtracted from total body energy to obtain the energy as protein. Liver triglycerides were measured by a colorimetric enzymic method using a commercial kit that utilizes lipoprotein lipase isolated from *Pseudomonas* sp. (SGM Italia, Roma, Italy). Liver ceramide content was evaluated by ELISA as reported previously [4]. In brief, hepatic lipids extracted with the Folch method [19] (70 μl in methanol) were adsorbed to well bottoms overnight at 4°C. Plates were blocked with 10 mM PBS, 140 mM NaCl, 0.1% Tween, pH 7.4, supplemented with 1% BSA for 1 h at 37°C. The plates were then washed three times with 10 mM PBS, 140 mM NaCl, 0.05% Tween, pH 7.4 (Tween-PBS), and incubated with monoclonal anti-ceramide antibody (2 μg/ml) for 1 h at 37°C. After three washes in Tween-PBS, peroxidase-conjugated goat anti-mouse immunoglobulin M (1:5000 dilution) was incubated for 1 h at 37°C. After four washes in Tween-PBS, the wells were incubated with 100 μl of a colour development solution (20 mg of *o*-phenylenediamine dihydrochloride in 50 ml of 70 mM Na_2_HPO_4_, 30 mM citric acid, pH 5, supplemented with 120 μl of 3% H_2_O_2_). After 15 min at 37°C, the reaction was stopped by the addition of 50 μl of 2.5 M H_2_SO_4_ and the absorbance was measured at 492 nm. All tests were carried out in triplicate. Immunoreactivity was normalized to the starting tissue weight. Negative control reactions omitted the primary antibody.

Energy balance measurements were conducted by the comparative carcass technique over the experimental period, as detailed previously [20]. In brief, daily food consumption was monitored, the energy density of the diet was measured by a bomb calorimeter, and energy intake was calculated. Faecal energy loss was determined from the energy measured in faeces. Body energy and lipid gain were calculated as the difference between the final content of body energy and fat, and the body energy and fat content of a group of rats that was killed at the beginning of the dietary treatment and used for the determination of initial body energy and lipid content.

### Myeloperoxidase (MPO) activity in ileum, liver, and v-WAT

The determination of MPO activity can be used as a surrogate marker of inflammation, since it has been shown that the activity of MPO solubilized from the inflamed tissue is directly proportional to the number of neutrophils seen in histological sections [21]. MPO activity was therefore assessed in ileum, v-WAT, and liver samples, as reported by Kim et al. [22]. In brief, tissue samples (100 mg) were homogenized in 1 ml of hexadecyltrimethylammonium bromide (HTAB) buffer (0.5% HTAB in 50 mM phosphate buffer, pH 6.0) and centrifuged at 13,400 × *g*_av_ for 6 min at 4°C. Then, 10 µl of supernatant was combined with 200 µl of 50 mM phosphate buffer, pH 6.0, containing 0.167 mg/ml *O*-dianisidine hydrochloride and 1.25% hydrogen peroxide. The change in absorbance at 450 nm was measured, and one unit of MPO activity was defined as that degrading 1 µmol of peroxide per minute at 25°C.

### Isolation of liver mitochondria, and measurement of mitochondrial oxidative capacities and degree of coupling

Isolation of liver mitochondria and measurement of state 3 respiration were carried out as previously reported [23]. In brief, liver tissue fragments were gently homogenized with a medium containing 220 mM mannitol, 70 mM sucrose, 20 mM 4-(2-hydroxyethyl)-1-piperazineethanesulfonic acid (HEPES), 1 mM EDTA, and 0.1% (w/v) fatty acid-free BSA, pH 7.4, in a Potter Elvehjem homogenizer set at 500 rpm (4 strokes/min). After withdrawal of aliquots for further assays, the homogenate was centrifuged at 1000 × *g*_av_ for 10 min and the resulting supernatant was again centrifuged at 3000 × *g*_av_ for 10 min. The mitochondrial pellet was washed twice and finally resuspended in a medium containing 80 mM KCl, 50 mM HEPES, 5 mM Tris–PO_4_, 1 mM ethylene glycol-bis(β-aminoethyl ether)-*N,N,N*',*N*'-tetraacetic acid (EGTA), and 0.1% (w/v) fatty acid-free BSA, pH 7.0. The oxygen consumption rate was measured polarographically with a Clark-type electrode (Yellow Springs Instruments, Yellow Springs, OH, USA) in a 3 ml glass cell, at a temperature of 30°C in a medium containing 80 mM KCl, 50 mM HEPES, 5 mM K_2_HPO_4_, 1 mM EGTA, and 0.1% (w/v) fatty acid-free BSA, pH 7.0. All samples were allowed to oxidize their endogenous substrates for 3 min and then succinate 10 mM plus rotenone 3.75 µM were added as substrate. State 3 oxygen consumption was measured in the presence of 0.3 mM adenosine diphosphate (ADP), while state 4 was obtained from oxygen consumption measurements at the end of state 3, when ADP becomes limiting. The respiratory control ratio (RCR) was calculated as the state 3/state 4 ratio, and the ADP/O ratio was calculated as the ratio between the moles of added ADP and the moles of oxygen consumed during state 3. Control experiments of enzymic and electron microscopic characterization showed that our isolation procedure (centrifugation at 3000 × *g*_av_ for 10 min) results in a cellular fraction that is constituted essentially of mitochondria.

The degree of coupling was determined in liver mitochondria by applying equation (11) of Cairns et al. [24]: Degree of coupling = 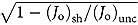, where (*J*_o_)_sh_ represents the oxygen consumption rate in the presence of oligomycin that inhibits ATP synthase, and (*J*_o_)_unc_ is the uncoupled rate of oxygen consumption induced by carbonyl cyanide 4-(trifluoromethoxy)phenylhydrazone (FCCP), which dissipates the transmitochondrial proton gradient. (*J*_o_)_sh_ and (*J*_o_)_unc_ were measured as above using succinate (10 mM) plus rotenone (3.75 µM) in the presence of oligomycin (2 µg/ml) or FCCP (1 µM), respectively.

### Hepatic stearoyl-coenzyme A desaturase (SCD1) activity, hepatic mitochondrial lipid peroxidation, and aconitase and superoxide dismutase (SOD)-specific activity

SCD1 activity was measured polarographically in liver homogenates at 37°C in a solution containing 0.1 M K_2_HPO_4_, pH 7.4, 1 µM myxothiazol, 0.12 mM NADH, and 0.06 mM stearoyl-coenzyme A as cyanide (5 mM)-sensitive [25], myxothiazol-insensitive oxygen consumption.

Lipid peroxidation was determined in isolated mitochondria using the same procedure as for plasma samples.

Active aconitase-specific activity was measured spectrophotometrically by following the formation of NADPH (340 nm) at 25°C in a mixture containing 0.2 mM NADP^+^, 5 mM sodium citrate, 0.6 mM MnCl_2_, 1 U/ml isocitric dehydrogenase, 50 mM Tris–HCl, pH 7.4, and 30 ml of mitochondrial extract [26]. Aconitase inhibited by reactive oxygen species (ROS) *in vivo* was reactivated so that total activity could be measured by incubating mitochondrial extracts in a medium containing 50 mM dithiothreitol, 0.2 mM Na_2_S, and 0.2 mM ferrous ammonium sulphate.

Mn-SOD-specific activity was measured in a medium containing 0.1 mM EDTA, 2 mM KCN, 50 mM KH_2_PO_4_, pH 7.8, 20 mM cytochrome *c*, 0.1 mM xanthine, and 0.01 units of xanthine oxidase. Determinations were carried out spectrophotometrically (550 nm) at 25°C, by monitoring the decrease in the reduction rate of cythocrome *c* by superoxide radicals, generated by the xanthine–xanthine oxidase system. One unit of SOD activity is defined as the concentration of enzyme that inhibits cythocrome *c* reduction by 50% in the presence of xanthine plus xanthine oxidase [27].

### Western blot quantification of occludin in ileum, serine palmitoyl-coenzyme A transferase (SPT) in liver, and p-Akt in liver and v-WAT

Tissue samples were denatured in a buffer (60.0 mmol/l Tris, pH 6.8, 10% sucrose, 2% SDS, 4% β-mercaptoethanol) and loaded on a 12% sodium dodecyl sulphate (SDS)–polyacrylamide gel. After the run in electrode buffer (50 mmol/l Tris, pH 8.3, 384 mmol/l glycine, 0.1% SDS), the gels were transferred on polyvinylidene difluoride (PVDF) membranes (Millipore, Billerica, MA, USA) at 0.8 mA/cm^2^ for 90 min. The membranes were preblocked in blocking buffer (PBS, 5% milk powder, 0.5% Tween 20) for 1 h and then incubated overnight at 4°C with polyclonal antibody for occludin (ThermoScientific, Schaumburg, IL, USA; diluted 1:250 in blocking buffer), SPT (Abcam, Cambridge, UK; diluted 1:300 in blocking buffer), or p-Akt (Cell Signaling, Danvers, MA, USA; diluted 1:1000 in blocking buffer). Membranes were washed three times for 12 min in PBS/0.5% Tween 20, and three times for 12 min in PBS, and then incubated for 1 h at room temperature with an anti-rabbit, alkaline phosphatase-conjugated secondary antibody (Promega, Madison, WI, USA). The membranes were washed as described above, rinsed in distilled water, and incubated at room temperature with a chemiluminescent substrate, CDP-Star (Sigma-Aldrich, St Louis, MO, USA). Data detection was carried out by exposing autoradiography films (Eastman Kodak Company, New York, NY, USA) to the membranes. Quantification of signals was carried out by Un-Scan-It gel software (Silk Scientific, Orem, UT, USA). Akt was detected with polyclonal antibody (Cell Signaling, Danvers, MA, USA; diluted 1:1000 in blocking buffer) and used to normalize the p-Akt signal, while actin was detected with polyclonal antibody (Sigma-Aldrich, St Louis, MO, USA; diluted 1:250 in blocking buffer) and used to normalize the occludin or SPT signal.

### 16S metagenomic sequencing of rat caecal content

The caecal content was squeezed out, collected separately, and immediately placed on dry ice. The total genomic DNA was extracted in triplicate from 200 mg of each caecal microbiota sample using the QIAamp® DNA Stool Mini Kit (Qiagen, Hilden, Germany), following the manufacturer’s instructions. The hypervariable regions V4–V5 of the 16S ribosomal RNA gene were amplified and sequenced as described before [10]. Uclust [28] was used to define operational taxonomic units (OTUs) at 97% sequence identity, which were assigned a taxonomy using the RDP classifier [29]. Representative sequences for each OTU were aligned with PyNast [30] and columns that were uninformative for phylogeny building were filtered out using Greengenes [31].

### Statistical analysis

Data are reported as means with their standard errors. Statistical analyses were performed by two-way analysis of variance (ANOVA) followed by Bonferroni post test. Probability values less than 0.05 were considered to indicate a significant difference. All analyses were performed using GraphPad Prism 6 (GraphPad Software, La Jolla, CA, USA).

### Materials

All chemicals used were of analytical grade and were purchased from Sigma (St Louis, MO, USA).

## Results

### Body composition and energy balance

Fructose-fed rats exhibited significantly higher body energy and lipids compared to controls, with no variation in body proteins (Table 2). Antibiotic treatment did not have any effect on these parameters (Table 2). Energy intake and faecal energy loss were similar in the four groups of rats, while energy and lipid gain were significantly higher in fructose-fed rats than in controls, with no effect of antibiotic treatment (Table 2).

**Table 2.** Body composition and energy balance.

	Control	Fructose	Control + antibiotic	Fructose + antibiotic	Two-way ANOVA *p*	
					Diet effect	Antibiotic effect
Initial body weight (g)	461 ± 12^a^	459 ± 9^a^	460 ± 10^a^	460 ± 10^a^	0.924	> 0.999
Final body weight (g)	535 ± 23^a^	541 ± 15^a^	531 ± 15^a^	539 ± 15^a^	0.691	0.864
Final body energy (kJ)	4455 ± 100^a^	5294 ± 120^b^	4473 ± 150^a^	5274 ± 130^b^	< 0.0001	0.994
Final body lipids (kJ)	2093 ± 100^a^	2759 ± 210^b^	2088 ± 120^a^	2749 ± 201^b^	0.0007	0.964
Final body proteins (kJ)	2462 ± 100^a^	2435 ± 110^a^	2496 ± 88^a^	2425 ± 200^a^	0.715	0.929
Energy intake (kJ)	25,700 ± 1235^a^	25,590 ± 388^a^	25,688 ± 878^a^	25,500 ± 336^a^	0.854	0.950
Faecal energy loss (kJ)	6002 ± 230^a^	6050 ± 201^a^	6098 ± 180^a^	6038 ± 200^a^	0.977	0.839
Energy gain (kJ)	966 ± 70^a^	1284 ± 60^b^	908 ± 70^a^	1277 ± 70^b^	< 0.0001	0.636
Lipid gain (kJ)	753 ± 70^a^	1015 ± 70^b^	703 ± 50^a^	1062 ± 85^b^	0.0002	0.983

Values are the mean ± SEM of six different rats. ANOVA: analysis of variance.

Values in the same row with different superscript letters are significantly different (*p* < 0.05, Bonferroni post test).

### Glucose homeostasis

**Figure 1.**
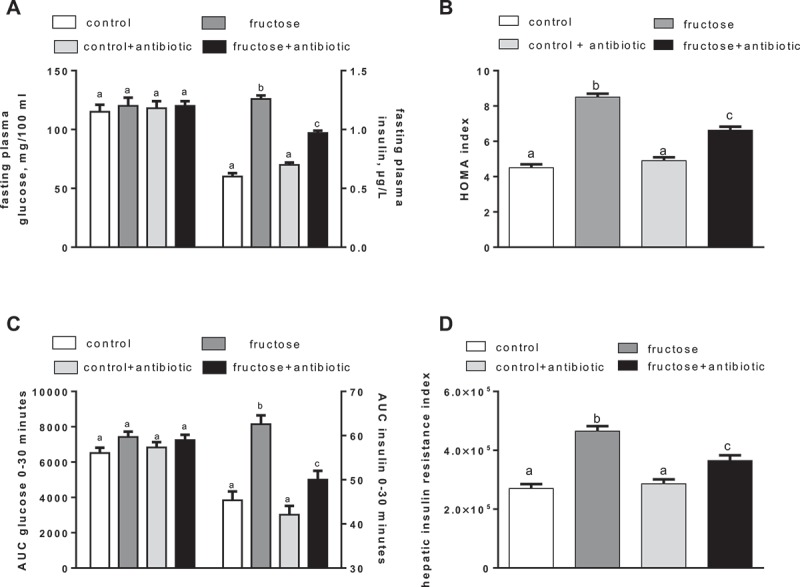
Parameters of glucose homeostasis in rats fed a control or fructose-rich diet, with or without antibiotic in the drinking water, after 4 weeks of treatment. Rats were food deprived, and after 6 h, plasma glucose and insulin (A) and homeostatic model assessment (HOMA) index (B) were determined. Then, glucose (2 g/kg body weight) was injected intraperitoneally, plasma glucose and insulin were measured after 15 and 30 min, and the area under the curve (AUC) for both parameters was calculated (C), together with the hepatic insulin resistance index, assessed as the product of (AUC glucose × AUC insulin) (D). Values are the mean ± SEM of six different rats. Values with different superscript letters are significantly different (*p* < 0.05, Bonferroni post test). Two-way analysis of variance *p* results: (A) fasting plasma insulin diet effect < 0.0001, antibiotic effect = 0.0013; (B) diet effect < 0.0001, antibiotic effect = 0.0015; (C) AUC insulin diet effect < 0.0001, antibiotic effect = 0.0116; (D) diet effect < 0.0001, antibiotic effect = 0.0007).

After 4 weeks of treatment, fructose-fed rats exhibited significantly higher values of fasting plasma insulin (Figure 1(A)) and HOMA index (Figure 1(B)) compared to control rats, while antibiotic treatment partly reversed these increases (Figure 1(A, B)). The results of the glucose tolerance test indicate that the insulin response during the first 30 min was significantly higher in fructose-fed rats than in controls (Figure 1(C)). In addition, the hepatic insulin resistance index, calculated during this early phase of the glucose tolerance test, was found to be significantly higher in fructose-fed rats (Figure 1(D)). Antibiotic treatment partly reversed the increase in insulin response during glucose load (Figure 1(C)) and in the hepatic insulin resistance index found in fructose-fed rats (Figure 1(D)).

**Figure 2.**
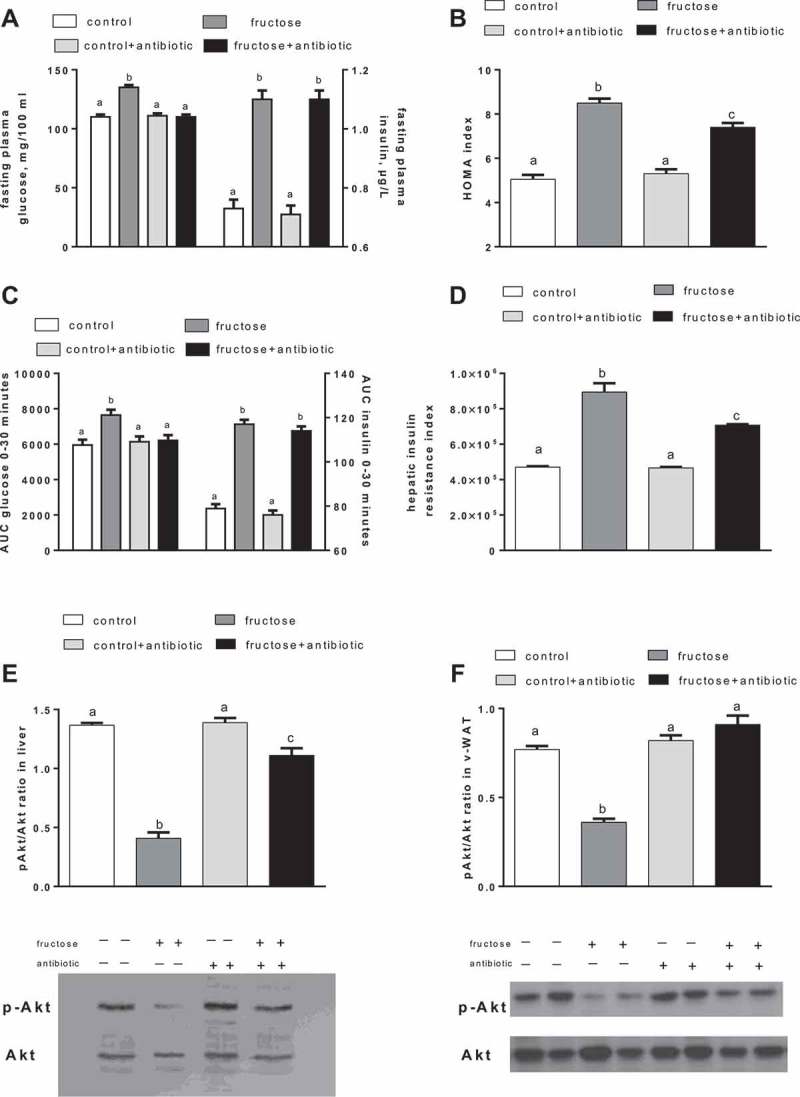
Parameters of glucose homeostasis in rats fed a control or fructose-rich diet, with or without antibiotic in the drinking water, after 8 weeks of treatment. Rats were food deprived, and after 6 h, plasma glucose and insulin (A) and homeostatic model assessment (HOMA) index (B) were determined. Then, glucose (2 g/kg body weight) was injected intraperitoneally, plasma glucose and insulin were measured after 15 and 30 min, and the area under the curve (AUC) for both parameters was calculated (C), together with the hepatic insulin resistance index (D). Insulin sensitivity was also measured in the liver and visceral white adipose tissue (v-WAT) by determination of the p-Akt/Akt ratio (E, F, with representative Western blots). Values are the mean ± SEM of six different rats. Values with different superscript letters are significantly different (*p < 0.05, Bonferroni post test). Two-way analysis of variance *p* results: (A) fasting plasma glucose diet effect < 0.0001, antibiotic effect < 0.0001; fasting plasma insulin diet effect < 0.0001, antibiotic effect = not significant; (B) diet effect < 0.0001, antibiotic effect = 0.026; (C) AUC glucose diet effect = 0.0079, antibiotic effect = 0.03; AUC insulin diet effect < 0.0001, antibiotic effect = not significant; (D) diet effect < 0.0001, antibiotic effect = 0.0007; (E) diet effect < 0.0001, antibiotic effect < 0.0001; (F) diet effect < 0.0001, antibiotic effect < 0.0001).

Significantly higher fasting values of plasma glucose and insulin (Figure 2(A)), as well as higher HOMA index values (Figure 2(B)), were found in fructose-fed rats, compared to controls, at the end of the treatment. Glucose and insulin (Figure 2(C)) response during the first 30 min following the glucose load were significantly higher in fructose-fed rats compared to controls. In addition, the hepatic insulin resistance index, calculated during this early phase of the glucose tolerance test, was found to be significantly higher in fructose-fed rats (Figure 2(D)). Antibiotic treatment abolished the increase in fasting plasma glucose (Figure 2(A)) and in the glucose response to glucose load (Figure 2(C)), and partly reversed the increase in the HOMA index (Figure 2(B)) and hepatic insulin resistance index (Figure 2(D)) found in fructose-fed rats. The degree of phosphorylation of the kinase Akt, a distal effector of insulin signalling, was found to be significantly lower in both the liver and v-WAT of fructose-fed rats, while antibiotic treatment reversed this effect, partly in liver and fully in v-WAT (Figure 2(E, F)).

### Ileum, liver, and v-WAT inflammation

**Figure 3.**
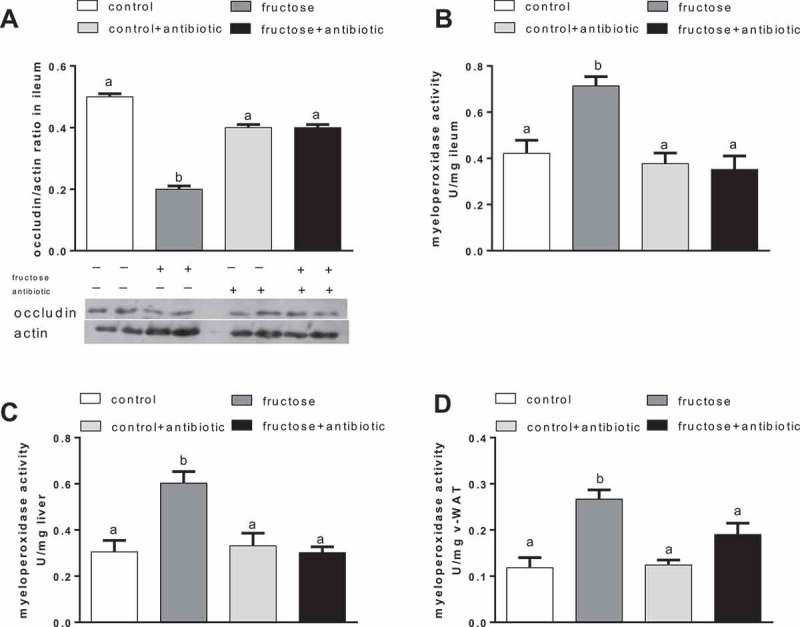
Occludin protein quantification in ileum (A) and myeloperoxidase activity in ileum (B), liver (C), and visceral white adipose tissue (v-WAT) (D) in rats fed a control or fructose-rich diet, with or without antibiotic in the drinking water, after 8 weeks of treatment. Values are the mean ± SEM of six different rats. Values with different superscript letters are significantly different (*p < 0.05, Bonferroni post test). Two-way analysis of variance *p* results: (A) diet effect < 0.0001, antibiotic effect < 0.0001; (B) diet effect = 0.012, antibiotic effect = 0.005; (C) diet effect = 0.0088, antibiotic effect = 0.0075; (D) diet effect < 0.0104, antibiotic effect = 0.031).

Quantification of occludin in the ileum revealed a significant fructose-induced decrease in this tight-junction protein, which was not evident in antibiotic-treated rats (Figure 3(A)). MPO is an enzyme produced in leucocytes, and its activity is linearly related to neutrophil infiltration of the tissues as an index of inflammatory response under pathological conditions [32]. Fructose-fed rats exhibited a significant increase in MPO activity in the ileum, liver, and v-WAT, while antibiotic treatment abolished this increase (Figure 3(B, C and –D)).

### Plasma parameters, hepatic lipid composition, and SCD-1 activity

In fructose-fed rats, plasma levels of ALT and AST, biochemical indicators of hepatic damage, and plasma levels of TBARS, markers of lipid peroxidation, were significantly higher than in controls, and antibiotic treatment of fructose-fed rats significantly lowered these increases (Table 3). Similarly, portal concentrations of TNF-a and LPS, as well as ileal TNF-α were significantly higher in fructose-fed rats than in controls, and antibiotic treatment of fructose-fed rats abolished these increases (Table 3). Plasma levels of triglycerides (Table 3) and hepatic triglycerides, and SCD-1 activity (Table 4) were significantly higher in fructose-fed rats compared to controls, and antibiotic treatment did not affect these parameters. Finally, a significant increase in hepatic levels of ceramide and TBARS was evident in fructose-fed rats, and antibiotic treatment reversed this effect, while no variation due to diet or antibiotic treatment was found in hepatic protein expression of SPT, the first and rate-limiting enzyme in ceramide synthesis [33] (SPT/actin ratio: 0.77 ± 0.02 in control rats; 0.85 ± 0.03 in fructose-fed rats; 0.80 ± 0.05 in control + antibiotic rats; 0.90 ± 0.04 in fructose-fed + antibiotic rats).

**Table 3.** Plasma parameters and portal inflammatory markers.

	Control	Fructose	Control + antibiotic	Fructose + antibiotic	Two-way ANOVA *p*	
					Diet effect	Antibiotic effect
Plasma triglycerides (mg/100 ml)	112 ± 4^a^	191 ± 5^b^	130 ± 5^a^	212 ± 7^b^	< 0.0001	0.002
Plasma ALT (U/l)	16.5 ± 1.0^a^	27.0 ± 1.0^b^	15.0 ± 1.0^a^	17.7 ± 1.0^a^	< 0.0001	< 0.0001
Plasma AST (U/l)	42.7 ± 3.0^a^	64.8 ± 3.0^b^	47.2 ± 2.0^a^	49.4 ± 2.0^a^	0.0001	0.04
Plasma lipid peroxidation (nmol TBARS/ml)	9.8 ± 0.9^a^	14.2 ± 0.5^b^	9.4 ± 0.5^a^	11.5 ± 0.5^a^	< 0.0001	0.027
Portal plasma NEFAs (mmol/l)	0.14 ± 0.01^a^	0.20 ± 0.01^b^	0.13 ± 0.01^a^	0.15 ± 0.01^a^	0.0007	0.0071
Portal plasma LPS (EU/ml)	0.563 ± 0.015^a^	0.980 ± 0.029^b^	0.514 ± 0.011^a^	0.562 ± 0.009^a^	< 0.0001	< 0.0001
Portal plasma TNF-α (pg/ml)	48.3 ± 3.2^a^	93.5 ± 5.1^b^	45.4 ± 3.0^a^	54.2 ± 2.6^a^	< 0.0001	< 0.0001
Ileum TNF-α (ng/mg protein)	8.7 ± 0.6^a^	14.9 ± 0.6^b^	10.3 ± 0.9^a^	9.4 ± 0.7^a^	0.0013	0.0125

Values are the mean ± SEM of six different rats.

ALT, alanine transaminase; AST, aspartate transaminase; TBARS, thiobarbituric acid reactive substances; NEFA, non-esterified fatty acid; LPS, lipopolysaccharide; EU, endotoxin unit; TNF-α, tumour necrosis factor-α; ANOVA, analysis of variance.

Values in the same row with different superscript letters are significantly different (*p* < 0.05, Bonferroni post test).

**Table 4.** Lipid composition and stearoyl-coenzyme A desaturase (SCD1) activity in liver.

	Control	Fructose	Control + antibiotic	Fructose + antibiotic	Two-way ANOVA *p*	
					Diet effect	Antibiotic effect
Triglycerides (mg/g)	14.9 ± 0.8^a^	21.9 ± 1.1^b^	15.0 ± 1.1^a^	21.2 ± 1.2^b^	< 0.0001	0.78
Ceramide (AU/g liver)	239 ± 27^a^	346 ± 21^b^	244 ± 11^a^	253 ± 20^a^	0.0106	0.045
Lipid peroxidation (nmol TBARS/g liver)	61.9 ± 2.1^a^	75.4 ± 2.0^b^	63.5 ± 2.0^a^	55.9 ± 1.9^a^	0.045	0.0002
SCD1 activity [ng atoms O/(min × g liver)]	98 ± 3^a^	175 ± 7^b^	102 ± 5^a^	203 ± 11^b^	< 0.0001	0.04

Values are the mean ± SEM of six different rats.

AU, absorbance unit; TBARS, thiobarbituric acid reactive substances; O, oxygen; ANOVA, analysis of variance.

Values in the same row with different superscript letters are significantly different (*p* < 0.05, Bonferroni post test).

### Respiratory capacities, ADP/O ratio, and degree of coupling in isolated liver mitochondria

Mitochondrial efficiency was assessed by measuring the ADP/O ratio and degree of coupling (Table 5), and both parameters were found to be significantly higher in fructose-fed rats compared to controls, while antibiotic treatment reversed the effect of the fructose-rich diet (Table 5). No effect of diet or antibiotics was found in state 3 and 4 respiratory capacities, or in RCR and FCCP-stimulated respiration (Table 5).

**Table 5.** Respiratory capacities, adenosine diphosphate/oxygen (ADP/O) ratio, and degree of coupling in isolated liver mitochondria.

	Control	Fructose	Control + antibiotic	Fructose + antibiotic	Two-way ANOVA *p*	
					Diet effect	Antibiotic effect
State 3 [ngatoms O/(min x mg protein)]	181.9 ± 12^a^	191.3 ± 2.3^a^	192.5 ± 2.3^a^	196.0 ± 2.1^a^	0.314	0.235
State 4 [ngatoms O/(min x mg protein)]	31.4 ± 0.8^a^	31.5 ± 1.1^a^	29.6 ± 1.1^a^	29.5 ± 1.2^a^	> 0.999	0.088
RCR	6.0 ± 0.2^a^	6.1 ± 0.2^a^	6.5 ± 0.1^a^	6.5 ± 0.2^a^	0.784	0.068
ADP/O ratio	1.70 ± 0.08^a^	1.98 ± 0.08^b^	1.67 ± 0.08^a^	1.75 ± 0.08^a^	0.036	0.120
State 4 + oligomycin [ng atoms O/(min × mg protein)]	24.5 ± 0.6^a^	20.5 ± 0.6^b^	24.4 ± 0.6^a^	24.7 ± 1.0^a^	0.019	0.010
+ FCCP [ng atoms O/(min × mg protein)]	228 ± 10^a^	226 ± 9^a^	222 ± 10^a^	223 ± 8^a^	0.958	0.633
Degree of coupling	0.943 ± 0.002^a^	0.956 ± 0.002^b^	0.943 ± 0.002^a^	0.941 ± 0.001^a^	0.006	0.0005

Values are the mean ± SEM of six different rats.

RCR, respiratory control ratio; FCCP, carbonyl cyanide 4-(trifluoromethoxy)phenylhydrazone; ANOVA, analysis of variance.

Values in the same row with different superscript letters are significantly different (*p* < 0.05, Bonferroni post test).

### Oxidative damage to lipids and proteins, and SOD-specific activity in liver mitochondria

Mitochondrial oxidative status was assessed, taking into account oxidative damage to lipids through the determination of lipid peroxidation, oxidative damage to proteins through the measurement of the marker enzyme aconitase, and antioxidant defence through the determination of SOD activity. A significant increase in oxidative damage to mitochondrial lipids and proteins, together with a significant decrease in the activity of the antioxidant enzyme SOD, was evident in fructose-fed rats, while antibiotic treatment reversed all of these effects (Table 6).

**Table 6.** Oxidative damage to lipid and proteins, and superoxide dismutase (SOD)-specific activity in liver mitochondria.

	Control	Fructose	Control + antibiotic	Fructose + antibiotic	Two-way ANOVA *p*	
					Diet effect	Antibiotic effect
Lipid peroxidation (nmol TBARS/mg protein)	0.56 ± 0.02^a^	0.73 ± 0.02^b^	0.63 ± 0.02^a^	0.60 ± 0.02^a^	< 0.0001	< 0.0001
Active aconitase (mU/mg protein)	2.10 ± 0.05^a^	1.72 ± 0.06^b^	2.08 ± 0.05^a^	2.11 ± 0.04^a^	0.002	0.002
Total aconitase (mU/mg protein)	3.20 ± 0.07^a^	3.31 ± 0.08^a^	2.91 ± 0.05^a^	3.15 ± 0.04^a^	0.011	0.0012
Active aconitase/total aconitase	0.69 ± 0.02^a^	0.52 ± 0.03^b^	0.70 ± 0.03^a^	0.70 ± 0.04^a^	0.012	0.006
Mn-SOD (U/mg protein)	111 ± 7^a^	83 ± 7^b^	110 ± 6^a^	103 ± 6^a^	0.0143	0.161

Values are the mean ± SEM of six different rats.

TBARS, thiobarbituric acid reactive substances; ANOVA, analysis of variance.

Values in the same row with different superscript letters are significantly different (*p* < 0.05, Bonferroni post test).

### Caecal microbiota

**Figure 4.**
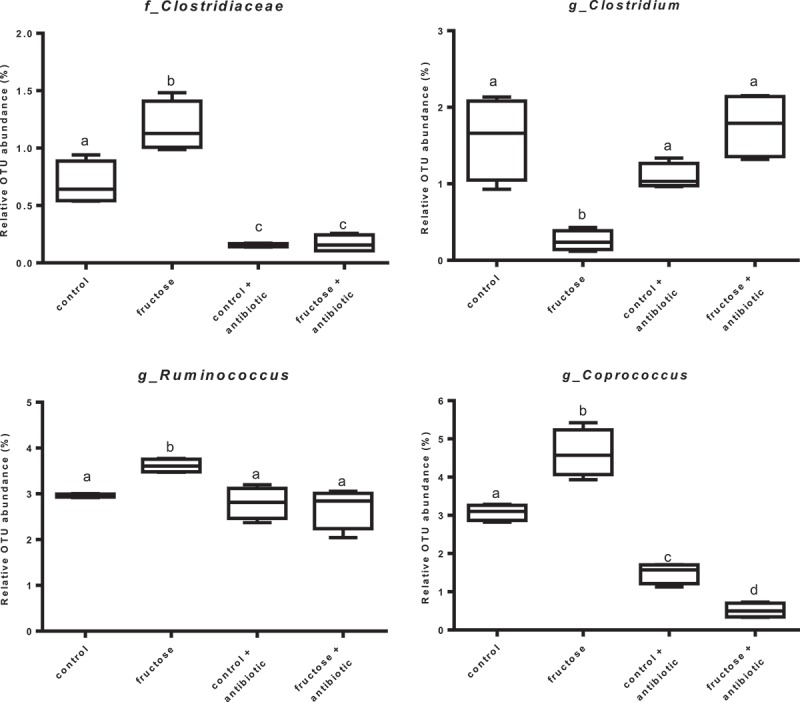
Representativeness of bacterial groups rescued by antibiotic treatment. Values are expressed as percentage of operational taxonomic units (OTU) abundance. Values are the mean ± SEM of six different rats. Values with different superscript letters are significantly different (*p < 0.05, Bonferroni post test). Two-way ANOVA *p* results: Clostridiaceae: diet effect = 0.0052, antibiotic effect < 0.0001; *Clostridium*: diet effect = 0.001, antibiotic effect = 0.0167; *Ruminococcus*: diet effect = 0.023, antibiotic effect = 0.0029; *Coprococcus*: diet effect = 0.013, antibiotic effect < 0.0001).

We have recently reported that a fructose-rich diet caused significant changes in the microbial composition of the caecum compared to the control group, and that antibiotic treatment caused a general reduction in microbial diversity [9]. Here, we performed a more detailed analysis at genus or family level to identify changes in caecal microbiota that were induced by a fructose-rich diet and reversed by antibiotic treatment. In particular, as shown in Figure 4, the representativeness of the genera *Coprococcus* and *Ruminococcus*, which was significantly increased by the fructose-rich diet, was restored by antibiotic treatment to levels similar to or even lower than those observed in the controls. The analysis of the Clostridiaceae family indicated that its representativeness was also significantly increased by the fructose-rich diet and restored by the antibiotic treatment. However, within this family the representativeness of the *Clostridium* genus was different, since it was reduced by the fructose-rich diet and restored to control levels by the antibiotic treatment (Figure 4).

## Discussion

In this study, we show that inflammation in the gut–liver–v-WAT axis, with defective insulin signalling and ceramide accumulation in the liver elicited by dietary fructose, can be reversed by modulation of the gut microbiota. When alterations in the gut microflora induced by a fructose-rich diet are prevented by antibiotic treatment, the above perturbations disappear.

Since the relationship between obesity and metabolic syndrome is well established [34], a great effort is being made to search for strategies that can control obesity and related diseases. Our present results show that it is possible to dissociate obesity from metabolic diseases, since we have found that antibiotic-treated rats show increased body lipids when fed a high-fructose diet, but do not exhibit the increase in the markers of inflammation found in fructose-fed rats not treated with antibiotics.

**Figure 5.**
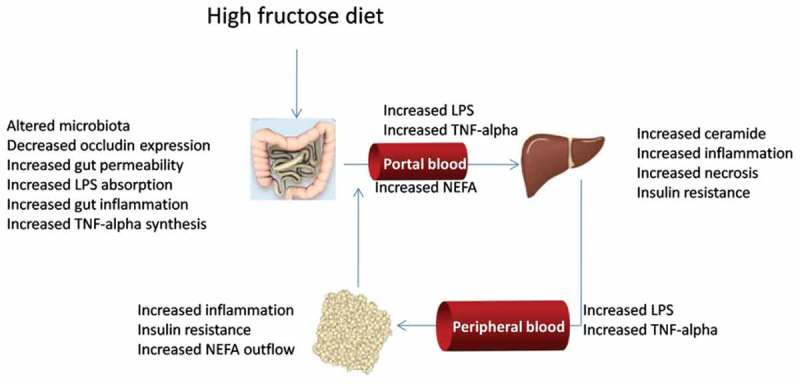
Gut–liver–visceral white adipose tissue axis interaction during high fructose feeding. LPS, lipopolysaccharide; TNF, tumour necrosis factor; NEFA, non-esterified fatty acids.

We have focused our attention on the portal circulation, since it links the gut, liver, and v-WAT. In particular, molecules coming from the gut and/or from v-WAT can influence liver metabolic activity. Our present result of a decreased content of the tight-junction protein occludin in the ileum of fructose-fed rats suggests that there is an increased gut permeability in these rats, thus allowing for higher translocation of LPS into the portal blood, in line with a previous proposal [35]. The higher portal levels of LPS in fructose-fed rats are, in turn, associated with an increased inflammatory status of the ileum in fructose-fed rats, evidenced by increased MPO activity and increased content of the proinflammatory cytokine TNF-α. The increased gut production of TNF-α then gives rise to increased portal TNF-α levels. The inflammatory mediators LPS and TNF-α could reach the liver through the portal circulatory system and exert their action on this tissue, probably through the activation of the pathway linked to Toll-like receptor-4, which has been found to be increased in mice fed a fructose-rich diet [36–38]. LPS and TNF-α then reach the systemic circulation [10] and in v-WAT they give rise to inflammation and insulin resistance, with a lower degree of phosphorylation of kinase Akt and increased outflow of NEFAs into the portal circulation. At the liver level, increased flux of NEFA and proinflammatory mediators generates inflammation, increased ceramide content, and insulin resistance (Figure 5). Antibiotic treatment in fructose-fed rats reduced inflammation in the ileum, abolished the increase in portal LPS and TNF-α, as well as the decrease in the ileal content of the tight-junction protein occludin, and reversed all the metabolic perturbations in the v-WAT and liver. It is of note that antibiotic treatment strongly reduced plasma oxidative stress and the derangement in glucose homeostasis, in spite of the same degree of obesity, fructose-induced hypertriglyceridaemia, and related increase in hepatic triglyceride deposition.

The abolished increase in hepatic ceramide in antibiotic-treated rats is in good agreement with the lower hepatic insulin resistance, and the higher degree of phosphorylation of kinase Akt, since it is well known that ceramide interferes with insulin signalling. The synthesis of ceramide can be stimulated by the increased flux of NEFAs to the liver, as well as by LPS, which up-regulates the synthesis of SPT, the rate-limiting enzyme of the ceramide biosynthetic pathway [33]. To discriminate between direct (through SPT regulation) and indirect (through regulation of NEFA outflow from v-WAT) effects of LPS, we assessed the liver content of SPT. The lack of any changes in the amount of SPT in the liver allows us to suggest that the increase in hepatic ceramide in fructose-fed rats is mainly due to an increased flux of NEFAs, and therefore is mainly dictated by inflammation in v-WAT.

The fructose-rich diet elicited the first signs of hepatic insulin resistance after only 4 weeks of dietary intervention, with fasting hyperinsulinaemia and an increased hepatic insulin resistance index. This metabolic impairment progressed further after 8 weeks, since hyperglycaemia during glucose load and fasting hyperglycaemia developed. The metabolic derangement in glucose homeostasis was linked to the gut microbiota, since in antibiotic-treated rats the hyperinsulinaemia and the increase in hepatic insulin resistance index found after 4 weeks of treatment were both significantly blunted, and the hyperglycaemic condition found after 8 weeks of treatment was completely prevented.

Fructose intake negatively affects liver function, since absorbed monosaccharides are first sent to the liver via portal blood, and up to 90% of ingested fructose is metabolized in the liver [39]. We have previously found that after long-term intake of a fructose-rich diet, the massive flow of fructose and its handling in the liver cause a metabolic stress to the tissue, evidenced by increased oxidative stress coupled with alterations of mitochondrial bioenergetic performance [3]. Therefore, another objective of our study was to investigate the extent to which manipulation of the gut microbiota through antibiotic treatment could be effective in the prevention of fructose-induced hepatic mitochondrial function. The issue is relevant since mitochondrial function and oxidative stress are key players in the development of liver injury. Accordingly, a fructose-rich diet deeply affects mitochondrial function by increasing the degree of coupling of oxidative phosphorylation and the ADP/O ratio. These modifications, in turn, could contribute, together with decreased antioxidant defence, to increased oxidative stress to lipids and proteins in hepatic mitochondria, as well as increased liver necrosis, as found in this study. An unwanted consequence of the increased degree of coupling is higher mitochondrial free radical production (ROS) [40,41]. The increased hepatic oxidative stress in fructose-fed rats could also be one of the factors leading to the onset of insulin resistance in this tissue [42,43]. Accordingly, the flavonoid naringenin has been found to prevent oxidative stress and reduce insulin resistance in the liver of fructose-fed rats [44], and the mitochondria-targeted antioxidant mitoQ has been shown to ameliorate features of the metabolic syndrome in obesogenic diet-fed rats, thus revealing the central role of mitochondrial oxidative stress in the development of the metabolic syndrome [45]. All of the above derangements are corrected when a fructose-rich diet is given to rats concomitantly with antibiotics, thus strongly suggesting that when alteration of the gut microbiota by a high-fructose diet is prevented, liver function is maintained.

Analysis of the caecal microbiota allowed us to identify at least four possible candidates involved in the metabolic link between a fructose-rich diet and hepatic derangement. *Ruminococcus*, *Coprococcus*, and members of the Clostridiaceae family, the abundance of which increased in fructose-fed rats, could favour fructose-induced liver disease; while the *Clostridium* genus, the abundance of which decreases in fructose-fed rats, could exert a protective effect on liver function. Increased *Ruminococccus* prevalence has been also found in obesity-prone rats [46] and in mice fed a high-fat diet [47], and increased *Ruminococccus* and *Coprococcus* prevalence has been found in obese men [48,49]. In addition, in rats fed a high-fat diet it has been found that Clostridiaceae abundance correlated with plasma insulin levels [50]. Thus, notwithstanding the differences in the animal model and dietary regimen used to elicit signs of the metabolic syndrome, the above members of the gut microbiota appear to play a relevant role in the induction of metabolic syndrome and related liver disease.

Taken together, our data show that a fructose-rich diet promotes alterations in the gut microbiota profile, associated with inflammation and metabolic dysregulation in the gut, liver, and v-WAT (Figure 5). These obesity-related features were reversed by changes in the gut microbiota profile induced by antibiotic therapy in fructose-fed rats. Our observations lead us to suggest that strategies that target modification of the gut microbiota profile could lead to benefits in preventing and attenuating obesity-related metabolic derangement.

## References

[1] Dekker MJ, Su Q, Baker C, et al Fructose: a highly lipogenic nutrient implicated in insulin resistance, hepatic steatosis, and the metabolic syndrome. Am J Physiol Endocrinol Metab. 2010;299(5):E685–E694. 20823452 10.1152/ajpendo.00283.2010

[2] Lyssiotis CA, Cantley LC. Metabolic syndrome: f stands for fructose and fat. Nature. 2013;502(7470):181–14. 24108049 10.1038/502181a

[3] Crescenzo R, Bianco F, Falcone I, et al Increased hepatic de novo lipogenesis and mitochondrial efficiency in a model of obesity induced by diets rich in fructose. Eur J Nutr. 2013;52:537–545. 22543624 10.1007/s00394-012-0356-y

[4] Crescenzo R, Bianco F, Coppola P, et al Increased skeletal muscle mitochondrial efficiency in rats with fructose-induced alteration in glucose tolerance. Br J Nutr. 2013;110:1996–2003. 23693085 10.1017/S0007114513001566

[5] Crescenzo R, Bianco F, Coppola P, et al Adipose tissue remodeling in rats exhibiting fructose-induced obesity. Eur J Nutr. 2014;53:413–419. 23728711 10.1007/s00394-013-0538-2

[6] Crescenzo R, Bianco F, Coppola P, et al Fructose supplementation worsens the deleterious effects of short-term high fat feeding on hepatic steatosis and lipid metabolism in adult rats. Exp Physiol. 2014;99(9):1203–1213. 24972835 10.1113/expphysiol.2014.079632

[7] Crescenzo R, Bianco F, Coppola P, et al The effect of high-fat-high-fructose diet on skeletal muscle mitochondrial energetics in adult rats. Eur J Nutr. 2015;54:183–192. 24743896 10.1007/s00394-014-0699-7

[8] Serino M, Luche E, Gres S, et al Metabolic adaptation to a high-fat diet is associated with a change in the gut microbiota. Gut. 2012;61:543–553. 22110050 10.1136/gutjnl-2011-301012 PMC3292714

[9] Payne AN, Chassard C, Lacroix C Gut microbial adaptation to dietary consumption of fructose, artificial sweeteners and sugar alcohols: implications for host–microbe interactions contributing to obesity. Obes Rev. 2012;13:799–809. 22686435 10.1111/j.1467-789X.2012.01009.x

[10] Di Luccia B, Crescenzo R, Mazzoli A, et al Rescue of fructose-induced metabolic syndrome by antibiotics or faecal transplantation in a rat model of obesity. PLoS One. 2015;10(8):e0134893. 26244577 10.1371/journal.pone.0134893 PMC4526532

[11] Bergheim I, Weber S, Vos M, et al Antibiotics protect against fructose-induced hepatic lipid accumulation in mice: role of endotoxin. J Hepatol. 2008;48(6):983–992. 18395289 10.1016/j.jhep.2008.01.035

[12] Carvalho BM, Guadagnini D, Tsukumo DM, et al Modulation of gut microbiota by antibiotics improves insulin signalling in high-fat fed mice. Diabetologia. 2012;5(10):2823–2834. 10.1007/s00125-012-2648-4 22828956

[13] Murphy EF, Cotter PD, Hogan A, et al Divergent metabolic outcomes arising from targeted manipulation of the gut microbiota in diet-induced obesity. Gut. 2013;62(2):220–226. 22345653 10.1136/gutjnl-2011-300705

[14] Membrez M, Blancher F, Jaquet M, et al Gut microbiota modulation with norfloxacin and ampicillin enhances glucose tolerance in mice. FASEB J. 2008;22(7):2416–2426. 18326786 10.1096/fj.07-102723

[15] Ferrier L, Berard F, Debrauwer L, et al Impairment of the intestinal barrier by ethanol involves enteric microflora and mast cell activation in rodents. Am J Pathol. 2006;168:1148–1154. 16565490 10.2353/ajpath.2006.050617 PMC1606551

[16] Cacho J, Sevillano J, de Castro J, et al Validation of simple indexes to assess insulin sensitivity during pregnancy in Wistar and Sprague-Dawley rats. Am J Physiol. 2008;295:E1269–E1276. 10.1152/ajpendo.90207.2008 18796548

[17] Abdul-Ghani MA, Matsuda M, Balas B, et al Muscle and liver insulin resistance indexes derived from the oral glucose tolerance test. Diab Care. 2008;30:89–94. 10.2337/dc06-1519 17192339

[18] Fernandes MA, Custódio JB, Santos MS, et al Tetrandrine concentrations not affecting oxidative phosphorylation protect rat liver mitochondria from oxidative stress. Mitochondrion. 2006;6:176–185. 16890028 10.1016/j.mito.2006.06.002

[19] Folch J, Lees M, Stanley GHS A simple method for the isolation and purification of total lipids from animal tissues. J Biol Chem. 1957;226:497–510. 13428781

[20] Crescenzo R, Bianco F, Falcone I, et al Hepatic mitochondrial energetic during catch-up fat after caloric restriction. Metab. 2010;59:1221–1230. 10.1016/j.metabol.2009.11.015 20045539

[21] Krawisz JE, Sharon P, Stenson WF Quantitative assay for acute intestinal inflammation based on myeloperoxidase activity. Assessment of inflammation in rat and hamster models. Gastroenterology. 1984;87:1344–1350. 6092199

[22] Kim JJ, Shajib MS, Manocha MM, et al Investigating intestinal inflammation in DSS-induced model of IBD. J Vis Exp. 2012;60:3678. 10.3791/3678 PMC3369627 22331082

[23] Crescenzo R, Bianco F, Falcone I, et al Mitochondrial energetic in liver and skeletal muscle after energy restriction in young rats. Br J Nutr. 2012;108:655–665. 22085624 10.1017/S0007114511005903

[24] Cairns CB, Walther J, Harken AH, et al Mitochondrial oxidative phosphorylation efficiencies reflect physiological organ roles. Am J Physiol. 1998;274:R1376–R1383. 9612405 10.1152/ajpregu.1998.274.5.R1376

[25] Strittmatter P, Spatz L, Corcoran D, et al Purification and properties of rat liver microsomal stearyl coenzyme A desaturase. Proc Natl Acad Sci USA. 1974;71:4565–4569. 4373719 10.1073/pnas.71.11.4565 PMC433928

[26] Gardner PR Aconitase: sensitive target and measure of superoxide. Meth Enzymol. 2002;349:9–16. 11912933 10.1016/s0076-6879(02)49317-2

[27] Flohè L, Otting F Superoxide dismutase assay. Meth Enzymol. 1974;105:93–104. 10.1016/s0076-6879(84)05013-8 6328209

[28] Edgar RC Search and clustering orders of magnitude faster than BLAST. Bioinformatics. 2010;26(19):2460–2461. 20709691 10.1093/bioinformatics/btq461

[29] Wang Q, Garrity GM, Tiedje JM, et al Naive Bayesian classifier for rapid assignment of rRNA sequences into the new bacterial taxonomy. Appl Environ Microbiol. 2007;73(16):5261–5267. 17586664 10.1128/AEM.00062-07 PMC1950982

[30] Caporaso JG, Bittinger K, Bushman FD, et al PyNAST: a flexible tool for aligning sequences to a template alignment. Bioinformatics. 2010;26(2):266–267. 19914921 10.1093/bioinformatics/btp636 PMC2804299

[31] DeSantis TZ, Hugenholtz P, Larsen N, et al Greengenes, a chimera-checked 16S rRNA gene database and workbench compatible with ARB. Appl Environ Microbiol. 2006;72(7):5069–5072. 16820507 10.1128/AEM.03006-05 PMC1489311

[32] Villegas I, de la Lastra CA, Orjales A, et al A new flavonoid derivative, dosmalfate, attenuates the development of dextran sulphate sodium-induced colitis in mice. Int Immunopharmacol. 2003;3:1731–1741. 14636824 10.1016/j.intimp.2003.07.002

[33] Pagadala M, Kasumov T, McCullough AJ, et al. Role of ceramides in nonalcoholic fatty liver disease. Trends Endocrinol Metab. 2012;23:365–371. 22609053 10.1016/j.tem.2012.04.005 PMC3408814

[34] Ritchie SA, Connell JMC The link between abdominal obesity, metabolic syndrome and cardiovascular disease. Nutr Metab Cardiovasc Dis. 2007;17:319–326. 17110092 10.1016/j.numecd.2006.07.005

[35] Spruss A, Bergheim I Dietary fructose and intestinal barrier: potential risk factor in the pathogenesis of nonalcoholic fatty liver disease. J Nutr Biochem. 2009;20:657–662. 19679262 10.1016/j.jnutbio.2009.05.006

[36] Spruss A, Kanuri G, Wagnerberger S, et al Toll-like receptor 4 is involved in the development of fructose-induced hepatic steatosis in mice. Hepatology. 2009;50:1094–1104. 19637282 10.1002/hep.23122

[37] Wagnerberger S, Spruss A, Kanuri G, et al Toll-like receptors 1–9 are elevated in livers with fructose-induced hepatic steatosis. Br J Nutr. 2012;107:1727–1738. 22018861 10.1017/S0007114511004983

[38] Sellmann C, Priebs J, Landmann M, et al Diets rich in fructose, fat or fructose and fat alter intestinal barrier function and lead to the development of nonalcoholic fatty liver disease over time. J Nutr Biochem. 2015;26:1183–1192. 26168700 10.1016/j.jnutbio.2015.05.011

[39] Tappy L, Le KA Metabolic effects of fructose and the worldwide increase in obesity. Physiol Rev. 2010;90:23–46. 20086073 10.1152/physrev.00019.2009

[40] Korshunov SS, Skulachev VP, Starkov AA High protonic potential actuates a mechanism of production of reactive oxygen species in mitochondria. FEBS Lett. 1997;416:15–18. 9369223 10.1016/s0014-5793(97)01159-9

[41] Mailloux RJ, Harper ME Uncoupling proteins and the control of mitochondrial reactive oxygen species production. Free Radic Biol Med. 2011;51(6):1106–1115. 21762777 10.1016/j.freeradbiomed.2011.06.022

[42] Rains JL, Jain SK Oxidative stress, insulin signaling, and diabetes. Free Radic Biol Med. 2011;50(5):567–575. 21163346 10.1016/j.freeradbiomed.2010.12.006 PMC3557825

[43] Bettaieb A, Vazquez Prieto MA, Rodriguez Lanzi C, et al (-)-Epicatechin mitigates high-fructose-associated insulin resistance by modulating redox signaling and endoplasmic reticulum stress. Free Radic Biol Med. 2014;72:247–256. 24746618 10.1016/j.freeradbiomed.2014.04.011 PMC4077617

[44] Kannappan S, Palanisamy N, Anuradha CV Suppression of hepatic oxidative events and regulation of eNOS expression in the liver by naringenin in fructose-administered rats. Eur J Pharmacol. 2010;645(1–3):177–184. 20655900 10.1016/j.ejphar.2010.07.015

[45] Feillet-Coudray C, Fouret G, Ebabe Elle R, et al The mitochondrial-targeted antioxidant MitoQ ameliorates metabolic syndrome features in obesogenic diet-fed rats better than Apocynin or Allopurinol. Free Radic Res. 2014;48(10):1232–1246. 25066801 10.3109/10715762.2014.945079

[46] Duca FA, Sakar Y, Lepage P, et al Replication of obesity and associated signaling pathways through transfer of microbiota from obese-prone rats. Diabetes. 2014;63:1624–1636. 24430437 10.2337/db13-1526

[47] Kim KA, Gu W, Lee IA, et al High fat diet-induced gut microbiota exacerbates inflammation and obesity in mice via the TLR4 signaling pathway. PLoS One. 2012;7(10):e47713. 23091640 10.1371/journal.pone.0047713 PMC3473013

[48] Ferrer M, Ruiz A, Lanza F, et al Microbiota from the distal guts of lean and obese adolescents exhibit partial functional redundancy besides clear differences in community structure. Env Microbiol. 2013;15(1):211–226. 22891823 10.1111/j.1462-2920.2012.02845.x

[49] Kasai C, Sugimoto K, Moritani I, et al Comparison of the gut microbiota composition between obese and non-obese individuals in a Japanese population, as analyzed by terminal restriction fragment length polymorphism and next-generation sequencing. BMC Gastroenterol. 2015;15:100. 26261039 10.1186/s12876-015-0330-2 PMC4531509

[50] Lecomte V, Kaakoush NO, Maloney CA, et al Changes in gut microbiota in rats fed a high fat diet correlate with obesity-associated metabolic parameters. PLoS One. 2015;10(5):e0126931. 25992554 10.1371/journal.pone.0126931 PMC4436290

